# Atypical recruitment of medial prefrontal cortex in autism spectrum disorders: An fMRI study of two executive function tasks

**DOI:** 10.1016/j.neuropsychologia.2008.03.025

**Published:** 2008-07

**Authors:** Sam J. Gilbert, Geoffrey Bird, Rachel Brindley, Christopher D. Frith, Paul W. Burgess

**Affiliations:** a Institute of Cognitive Neuroscience and Department of Psychology, University College London, London, UK; b Wellcome Department of Imaging Neuroscience, Institute of Neurology, University College London, London, UK; c Center for Functional Integrative Neuroscience, University of Aarhus, Denmark

**Keywords:** Autism, Asperger syndrome, Executive function, fMRI, Prefrontal cortex

## Abstract

Recent studies have suggested an uneven profile of executive dysfunction in autism spectrum disorders (ASD). For example, some authors have reported deficits on newly developed tests of executive function sensitive to rostral prefrontal function, despite spared, or even superior, performance on other tests. We investigated the performance of a group of high-functioning participants with ASD (*N* = 15) and an age- and IQ-matched control group (*N* = 18) on two executive function tests, whilst undergoing functional magnetic resonance imaging (fMRI). Behaviourally, there were no significant differences between the two groups. In a classical test of executive function (random response generation), BOLD signal differed between the groups in the cerebellum but not in the frontal lobes. However, on a new test of executive function (selection between stimulus-oriented and stimulus-independent thought), the ASD group exhibited significantly greater signal-change in medial rostral prefrontal cortex (especially Brodmann Area 10) in the comparison of stimulus-oriented versus stimulus-independent attention. In addition, the new test (but not the classical test) provided evidence for abnormal functional organisation of medial prefrontal cortex in ASD. These results underline the heterogeneity of different tests of executive function, and suggest that executive functioning in ASD is associated with task-specific functional change.

Along with well-documented abnormalities in social interaction, communication, perception and attention (Frith, 2003), a large number of recent studies have provided evidence for disruption of executive functions in autism spectrum disorders (ASD; see e.g. Hill, 2004a,b; Ozonoff, Pennington, & Rogers, 1991; Ozonoff & Jensen, 1999; Russell, 1997; Russo et al., 2007). Functional neuroimaging studies have also pointed towards atypical brain activity in participants with ASD when performing executive function tasks (e.g. Just, Cherkassky, Keller, Kana, & Minshew, 2007; Luna et al., 2002; Schmitz et al., 2006). However, evidence from different studies has not always been consistent. In part, this mixed evidence may be accounted for by methodological issues such as the heterogeneity of the patient groups studied and the most appropriate ways of matching patient with control groups (Hill, 2004a,b). However, another explanation may be the heterogeneity of the processes referred to as ‘executive functions’ (Hill & Bird, 2006). Executive function is an umbrella term encompassing a wide range of high-level processes for controlling and organising behaviour, such as planning, inhibition, multitasking, monitoring and so on (Burgess, 1997; Gilbert & Burgess, 2008; Monsell, 1996; Shallice, 1988; Stuss & Knight, 2002). There is therefore no reason to suppose that performance on one test of executive function should necessarily mirror performance on another. Indeed, correlational studies have suggested that although there are often significant correlations between scores on various tests of executive function, these correlations tend to be rather low (typically *r* < 0.4; Duncan, Johnson, Swales, & Freer, 1997; Obonsawin et al., 2002). Moreover, factor analysis reveals the presence of multiple distinct factors in scores derived from batteries of executive function tests (e.g. Burgess, Alderman, Evans, Emslie, & Wilson, 1998; Miyake, Friedman, Emerson, Witzki, & Howerter, 2000).

Although the frontal lobes, and particularly the prefrontal cortex, have long been recognised as playing an important role in higher-level control (e.g. Luria, 1966; Penfield & Evans, 1935; Shallice, 1982), only recently have neuroimaging and neuropsychological studies begun to delineate distinct regions of prefrontal cortex supporting different aspects of executive function. In part, the evidence for such distinctions originated from studies of patients with frontal lobe lesions, who experienced behavioural disorganisation in everyday life with such severity that they were unable to return to work at their previous level, yet performed well on classical tests of executive function such as the Stroop task (Stroop, 1935), Wisconsin Card Sorting Test (WCST; Grant & Berg, 1948), Tower of London (Shallice, 1982), verbal fluency (Benton, 1968) and so on. Shallice and Burgess (1991) designed two new tasks – the “Multiple Errands Test” and “Six Element Test” – that were sensitive to deficits in three patients with frontal lobe lesions, who performed other tests of executive function within normal limits. Subsequent studies have suggested that these tasks, which depend upon behavioural organisation within relatively ill-constrained or ill-structured situations (i.e. multitasking), are particularly sensitive to lesions within the rostral prefrontal cortex, approximating Brodmann Area (BA) 10 (Burgess, 2000; Burgess, Veitch, Costello, & Shallice, 2000; see also Goel & Grafman, 2000).

Intriguingly, recent evidence suggests that executive deficits in high-functioning adults with ASD may be particularly apparent in new tests of executive function involving multitasking, rather than more constrained classical tests of executive function. Hill and Bird (2006) tested 22 high-functioning participants with ASD and 22 well-matched controls (matched on an individual basis) on a battery of executive function tests. Although there were no significant differences between the groups on standard tests of executive function (e.g. Stroop, WCST, verbal fluency), newer tests did distinguish the two groups, with abnormal behaviour in the ASD group being particularly apparent on the Six Element Test (SET).

Given the variability in results from previous studies (Hill, 2004a,b) it may be premature to point to a particular executive function test, or set of tests, that best distinguish participants with ASD from control participants. However, a consistent finding has been that participants with ASD tend to show deficits on only a restricted set of executive function tests, with preserved or superior performance in other domains (Hill & Bird, 2006; Minshew, Goldstein, & Siegel, 1997). This contrasts with other populations, who may be more likely to show more widespread deficits, such as those with schizophrenia (Bilder et al., 2000). The uneven profile of performance seen in studies that have investigated executive functions in ASD argues against a “deficit model” of the processing differences between participants with ASD and controls, and instead suggests that ASD may be better characterised by disruption or reorganisation of specific brain systems, rather than more generalised impairment (Minshew et al., 1997).

One candidate brain region that may show such disruption or reorganisation in ASD is medial rostral PFC, corresponding approximately to the medial part of Brodmann Area 10 and the adjacent “paracingulate” region (Frith & Frith, 2003). This region has been implicated in both structural (Abell et al., 1999) and functional (Castelli, Frith, Happé, & Frith, 2002) change in ASD (see Schmitz et al., 2006 for further evidence from a study that combined structural and functional imaging approaches). Furthermore, it has been argued that this area is involved in multitasking (Burgess et al., 2000; Burgess, Dumontheil, Gilbert, Okuda, et al., 2007) and mentalizing, or theory of mind (Frith & Frith, 2003), both of which have been suggested to be compromised in ASD (Hill & Bird, 2006; Baron-Cohen, Leslie, & Frith, 1985).

Recently, Burgess, Simons, Dumontheil, and Gilbert (2005) and Burgess, Dumontheil, and Gilbert (2007) have argued that particular regions of rostral PFC play an important role in selection between stimulus-oriented and stimulus-independent thought, i.e. attentional selection between current perceptual input versus self-generated information. Several recent neuroimaging studies have suggested that this form of attentional selection is supported by rostral PFC (Gilbert, Frith, & Burgess, 2005; Gilbert, Simons, Frith, & Burgess, 2006a; Gilbert, Williamson, et al., 2007). For example, in a study by Gilbert et al. (2005), participants performed three separate tasks that could either be accomplished by attending to task-relevant visual information (during “stimulus-oriented phases”), or by performing the same task “in their heads” (during “stimulus-independent phases”). Consistently across the three tasks, medial rostral PFC (approximating BA 10) showed greater activity throughout stimulus-oriented phases, compared with stimulus-independent phases. This finding is of particular relevance for understanding the brain mechanisms supporting multitasking. Tests of multitasking depend heavily on the ability to organise behaviour according to previously formed plans that are not immediately cued by the environment (i.e. prospective memory). For instance, in the SET, participants must perform the individual subtasks whilst at the same time maintaining an intention to switch between tasks in the future. In such situations, participants must bear in mind an internally represented intention to act whilst also monitoring events in the external environment as part of the ongoing tasks. It therefore seems likely that multitasking will depend critically on the ability to flexibly deploy attention between the external environment and internal representations (Burgess, Dumontheil, Gilbert, Okuda, et al., 2007). On this evidence, it seems possible that executive tasks stressing this form of attentional selection may be particularly sensitive to atypical information processing in ASD.

In the present study, we therefore investigated performance of participants with ASD performing two tests of executive function: one that is a more standard test, and one that requires switching between stimulus-oriented and stimulus-independent thought. For the standard test of executive function we chose a random response generation task. Random generation tasks have a long history of investigation within cognitive psychology (e.g. Baddeley, 1966). Such tasks place demands on executive functions in at least two respects. First, random generation may require participants to switch flexibly between different sequence-generation strategies (because using the same strategy for too long would lead to stereotyped responding). Second, it may be necessary to inhibit prepotent response tendencies, such as sequential cycling through the response options (Baddeley, Emslie, Kolodny, & Duncan, 1998). Neuroimaging studies have indicated widespread prefrontal and premotor activity associated with random generation tasks (e.g. Jahanshahi, Dirnberger, Fuller, & Frith, 2000). In addition, it has been shown that disruption of dorsolateral PFC activity with transcranial magnetic stimulation leads to an increase in stereotypical responding in such tasks (Jahanshahi et al., 1998). However, random generation tasks are not believed to depend critically on the functions of medial rostral PFC. The second executive function test used in the present study was a version of one of the tasks investigated by Gilbert et al. (2005), involving selection between stimulus-oriented and stimulus-independent thought. This task may index cognitive processes that also play a role in more complex situations, such as those involving prospective memory or behavioural organisation in ill-structured circumstances (Burgess, Dumontheil, Gilbert, Okuda, et al., 2007). We expected activity in medial rostral PFC to be particularly associated with the stimulus-oriented phases of this task.

## 1 Methods

### 1.1 Participants

Thirty-three individuals participated in the study: 15 participants with Autism Spectrum Disorder (12 males; 3 females) and 18 non-autistic control participants (13 males; 5 females). Groups were matched on age (ASD *M*: 38 years, SD: 13; control *M*: 32 years, SD: 8; *t*(31) = 1.6, *p* = .13), and IQ (ASD *M*: 119, SD: 14; control *M*: 119, SD: 11; *t*(31) = 0.1, *p* = .93). Full-scale IQ was measured using the Wechsler Adult Intelligence Scale 3rd UK Edition (Wechsler, 1999), apart from one control participant for whom IQ was estimated from the National Adult Reading Test (NART; Nelson, 1976). All participants in the ASD group had previously received a diagnosis from an independent clinician according to standard criteria. The Autism Diagnostic Observational Schedule-Generic (ADOS-G, Lord et al., 2000), was used to characterise the participants’ level of current functioning. This measure was chosen because all participants were adults; it was therefore not always possible to interview parents or caregivers, as required by other measures such as the Autism Diagnostic Interview (ADI; Lord, Rutter, & Le Couteur, 1994). On the ADOS-G, eight participants met criteria for autism, whilst six participants met criteria for autistic spectrum disorder. One participant was unwilling to complete the ADOS-G. All participants were right-handed, had normal or corrected-to-normal vision and were naïve with respect to the purpose of the experiment. None had performed the present experimental tasks, or related tasks, previously. The experiment was performed with local ethical committee approval and in accordance with the ethical standards laid down in the 1964 Declaration of Helsinki. Written informed consent was obtained from all participants before their inclusion in the study.

### 1.2 Tasks

*Random generation task:* In this task, participants were presented with a timing signal in the form of a small square (approximately 0.2° tall and wide), which appeared for 100 ms either every 750 ms (fast condition) or every 2000 ms (slow condition; see below). Participants were instructed to press one of four response buttons (with index or middle fingers of left or right hand) in synchrony with the appearance of this timing signal. In the experimental condition, participants were instructed to press buttons in a random sequence, as if the button-press on each trial were determined by rolling a die. In the baseline condition, participants repeatedly pressed the four buttons in a stereotyped sequence: left middle, left index, right index, right middle. The timing signal was presented in a different colour (green or blue) depending on condition, counterbalanced between participants.

*Alphabet task:* In this task (modified from Gilbert et al., 2005, Task 3) the experimental condition alternated between stimulus-oriented (SO) and stimulus-independent (SI) phases. During SO phases, participants classified capital letters by pressing one of two buttons, according to whether the letter was composed entirely of straight lines (e.g. “A”), or whether it had any curves (e.g. “B”). Immediately following each button press, the subsequent letter in the alphabet was presented. Stimuli were presented in Arial typeface, approximately 1° tall and wide. During SI phases, randomly chosen letters were presented and participants were required to mentally continue the sequence from their current position in the alphabet, performing the same classification task for each self-generated letter. In this condition, the correct continuation of the alphabet sequence was never presented on screen. Stimuli in the two phases were presented in different colours (red or blue), with the assignment of each colour to a particular phase counterbalanced across participants. The first letter to be presented in each SO phase was the appropriate continuation of the sequence, assuming that the sequence had been correctly maintained during the preceding SI phases. Transitions between the SO and SI phases occurred with a mean interval of 7.5 s (range 3–21 s). During blocks of the Alphabet task, participants also performed a baseline condition in which non-alphanumeric non-meaningful stimuli were presented, requiring classification as straight or curved. However, since the main purpose of the Alphabet task was to investigate selection between stimulus-oriented and stimulus-independent processing, this baseline condition (involving stimulus-oriented processing alone) is not examined further in the present article (for discussion see Gilbert, Bird, Frith, & Burgess, in preparation).

### 1.3 Scanning procedure

Participants were familiarised with the tasks during a practice session lasting approximately 15 min, immediately before the scanning session. A 3T Siemens Allegra head-only system was used to acquire both T1-weighted structural images and T2*-weighted echoplanar (EPI) images [64 × 64; 3 mm × 3 mm pixels; echo time (TE), 30 ms] with BOLD contrast. Each volume comprised 48 axial slices (2 mm thick, separated by 1 mm), oriented approximately parallel to the AC–PC plane, covering the whole brain. Functional scans were acquired during four sessions, each comprising 121 volumes (lasting ∼6 min). Volumes were acquired continuously with an effective repetition time (TR) of 3.12 s per volume. The first five volumes in each session were discarded to allow for T1 equilibration effects. Each task was performed for two of the four sessions, in an AABB order counterbalanced across participants. Within each session, participants alternated between experimental (40 s) and baseline (20 s) conditions, performing each condition five times per session. In the Random task, the timing signal was presented every 750 ms in one session, and every 2000 ms in the other, with the order counterbalanced between participants. Following the functional scans, a 12-min T1-weighted structural scan was performed.

### 1.4 Data analysis

fMRI data were analysed using SPM2 software (http://www.fil.ion.ucl.ac.uk/spm/spm2.html). Volumes were realigned, corrected for different slice acquisition times, normalized into 2 mm cubic voxels using a standard EPI template based on the Montreal Neurological Institute (MNI) reference brain using 4th-degree B-spline interpolation, and smoothed with an isotropic 8 mm full-width half-maximum Gaussian kernel. The volumes acquired during the four sessions were treated as separate time series. For each series, the variance in the BOLD signal was decomposed with a set of regressors in a general linear model (Friston et al., 1995). In the Alphabet task, variance was decomposed into components associated with responses made during the stimulus-oriented and stimulus-independent phases, as well as responses made during performance of the baseline straight/curved task. These regressors were derived from delta functions convolved with a canonical haemodynamic response function. An additional regressor indexed sustained activity during the instruction periods, using a boxcar function convolved with a canonical haemodynamic response function. In the Random task, variance was decomposed into components associated with sustained activity during the random generation phases and the baseline phases, using boxcar functions convolved with a canonical haemodynamic response function. These regressors, together with regressors representing residual movement-related artefacts and the mean over scans, comprised the full model for each session. The data and model were high-pass filtered to a cut-off of 1/128 Hz.

Parameter estimates for each regressor were calculated from the least mean squares fit of the model to the data. Effects of interest were assessed in random effects analyses using *t*-tests on contrast images generated from subject-specific analyses. Contrasts were thresholded at *P* < 0.001 uncorrected for multiple comparisons, with a minimum extent of five contiguous voxels.

## 2 Results

### 2.1 Behavioural data: random generation task

**Fig. 1:**
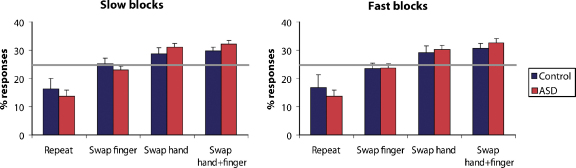
Behavioural data from the random generation task, presented separately for Slow and Fast blocks. Graphs indicate the percentage of responses of each of the four types, depending on the previous response. Horizontal lines indicate the expected percentage of responses in a truly random sequence. Error bars indicate standard errors.

The randomness of sequences produced in the experimental condition was evaluated by investigating dependencies between successive responses. In a truly random sequence, the probability of each of the four responses should be the same, regardless of the preceding response. However, previous studies have identified a number of biases in random generation behaviour, one of the most pervasive being the tendency of participants to avoid repeating their previous response (Falk & Konold, 1997). Fig. 1 shows the probability of each of the four possible responses – (a) same as last trial; (b) same hand, different finger; (c) other hand, same finger; d) other hand, different finger – separately for the ASD and control group, split into “fast” and “slow” blocks. Due to technical problems, behavioural data were not available in this task for two control participants. Both groups tended to repeat responses less often than would be expected by chance, and to swap hands more often. However, an ANOVA with within-subject factors of Response-Type and Speed, and a between-subject factor of Group (ASD, Control) showed that although there were significant deviations from truly random behaviour (*F*(3, 27) = 11.7; *p* < .0001), the groups did not differ in this respect (*F* < 1) and there were no interactions involving the Speed factor (*F*(3, 27) < 2.1, *p* > .12).

### 2.2 Behavioural results: alphabet task

**Fig. 2:**
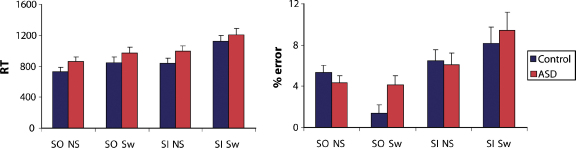
Behavioural data from the alphabet task. Left graph shows mean response time (RT) and right graph shows mean error rate, in each of four conditions depending on whether the phase was stimulus-oriented (SO) or stimulus-independent (SI), and whether the trial-type was switch (i.e. different phase to the previous trial: Sw) or non-switch (NS). Error bars indicate standard errors.

Mean RTs and error rates are displayed in Fig. 2. Mean RTs were examined in an ANOVA with within-subject factors Phase (Stimulus-Oriented [SO] or Stimulus-Independent [SI]) and Switch (Switch trial – i.e. immediately following a switch between the SO and SI phases – or Non-switch trial) and between-subjects factor Group (ASD or Control). There were main effects of Phase, SI trials being slower than SO trials, and Switch, Switch trials being slower than Non-switch trials, along with a Phase × Switch interaction, because the RT difference between Switch and Non-switch trials was larger in the SI phase than the SO phase (*F*(1, 31) > 21; *p* < .0001). However, although there was a trend towards slower RTs in the ASD group, neither the main effect of Group nor any of its interactions approached significance (*F*(1, 31) < 1.7, *p* > .2). A similar analysis of error rates revealed a main effect of Phase and a Phase × Switch interaction of the same type as the RT data (*F*(1, 31) > 9.7; *p* < .005). However, again there was no significant main effect or any significant interactions involving the Group factor (*F*(1, 31) < 4.1), *p* > .05).

### 2.3 fMRI results: random generation task

**Fig. 3:**
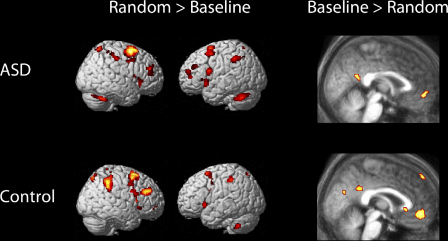
Signal change associated with the comparisons between Random and Baseline conditions, presented separately for the Control and ASD groups. Areas of activation in the contrasts of Baseline > Random are presented on sagittal slices (*x* = 2) of the relevant group of participants’ mean normalized structural scan.

**Fig. 4:**
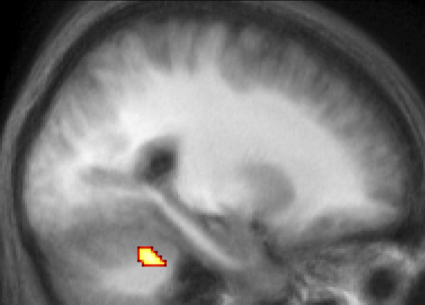
Regions showing significantly greater activation related to the contrast of Baseline > Random in the control than the ASD group. Results are plotted on a sagittal slice (*x* = −24) of the participants’ mean normalized structural scan.

Table 1 lists regions exhibiting significant differences in BOLD signal between the Random and Baseline conditions, separately for the ASD and Control groups (see also Fig. 3). Results were collapsed over the fast and slow blocks in this task, because there were no significant group differences associated with this factor. Both groups showed significantly increased signal during the Random condition in lateral PFC, premotor cortex/SMA, superior parietal cortex and cerebellum. In the reverse contrast (Baseline > Random), both groups showed differences in BOLD signal in medial rostral PFC, posterior cingulate and lateral temporal regions (Table 2). However, the only regions to show a significant difference between the ASD and control groups were left cerebellum and a small cluster of voxels in left lateral temporal cortex (BA 37), both of which showed a larger effect in the Baseline > Random contrast in the Control than the ASD group (Fig. 4; cerebellum: −24, −46, −30; *z* = 3.79; 108 voxels; temporal cortex: −42, −58, −18; *z* = 3.22; 6 voxels). Thus, both groups showed similar patterns of results in the frontal lobes: increased activity in the Random condition in lateral prefrontal regions and posterior frontal regions (SMA/anterior cingulate) and increased activity in the Baseline condition in medial rostral PFC (BA 10).

### 2.4 fMRI results: alphabet task

**Fig. 5:**
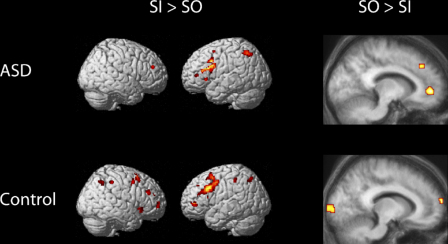
Signal change associated with the comparisons between stimulus-oriented (SO) and stimulus-independent (SI) conditions, presented separately for the Control and ASD groups. Areas of activation in the contrasts of SO > SI are presented on sagittal slices (*x* = −12) of the relevant group of participants’ mean normalized structural scan.

Table 3 lists regions exhibiting significant differences in BOLD signal between the Stimulus-Independent (SI) and Stimulus-Oriented (SO) conditions, separately for the ASD and Control groups (see also Fig. 5). Both groups showed significantly increased signal during the SI condition in lateral prefrontal and premotor regions, along with superior parietal cortex. However, of more theoretical interest was the analysis of regions showing increased signal in the SO condition, because this was the contrast expected to produce activity in medial rostral PFC, on the basis of previous studies (e.g. Gilbert et al., 2005; Gilbert, Simons, et al., 2006). This contrast produced activity in medial rostral PFC in both groups, along with lateral parietal cortex (Table 4). However, the medial PFC activation in the ASD group appeared more extensive than in the control group. This appearance was confirmed in a direct comparison between the groups, revealing significantly greater activity in the ASD group associated with the SO > SI contrast in medial PFC, along with temporal pole, amygdala, cerebellum, and other temporal and parietal regions (Table 5). Thus, whereas the random generation task did not reveal any frontal lobe differences between the groups, there was a significant difference between the groups in medial rostral PFC associated with the SO > SI contrast in the alphabet task. In addition, the control group exhibited significantly greater medial occipital and medial parietal activation associated with the SO > SI contrast than the ASD group (Table 5).

In order to test whether the apparently task-specific group differences reported above could reflect a thresholding artefact, rather than any genuine difference between the random generation and alphabet tasks, an analysis was conducted of Task × Group interactions. Whereas the chief between-group difference in the random generation task was observed in the cerebellum, the alphabet task revealed between-group differences primarily in medial rostral PFC. Both of these regions showed significant activity in the analysis of Task × Group interactions (e.g. cerebellum: −28, −44, −30; *z* = 3.31; 20 voxels; *p* < .0005; medial rostral PFC: 20, 60, 2; *z* = 3.36; 7 voxels; *p* < .0004; 12, 52, 34; *z* = 3.31; 153 voxels; *p* < .0005). Thus, the present results do not simply reflect the two tasks activating similar brain regions, with different regions just above or just below threshold.

In an additional analysis, the theoretically important group difference in medial rostral PFC was investigated at a more strict threshold, in order to confirm the present results after correcting for multiple comparisons across voxels. First, the control and ASD groups were collapsed, and the contrast of SO > SI was investigated in the alphabet task. At a threshold of *p* < .05, corrected for multiple comparisons across the whole brain, there was just a single region of activation (medial rostral PFC: 2, 64, 28; *z* = 4.71; *p*_corrected_ < .05). We then investigated regions responding more strongly to the SO > SI contrast in the ASD than the control group. No activations survived a whole-brain corrected threshold, but the group difference in medial rostral PFC was significant after correcting for multiple comparisons across a 20 mm sphere centred on the peak from the earlier orthogonal contrast of SO > SI, collapsed across the groups (10, 54, 36; *z* = 3.94; *p*_corrected_ < .025). Thus, the group difference in medial rostral PFC associated with the alphabet task was confirmed, even at a corrected threshold.

**Fig. 6:**
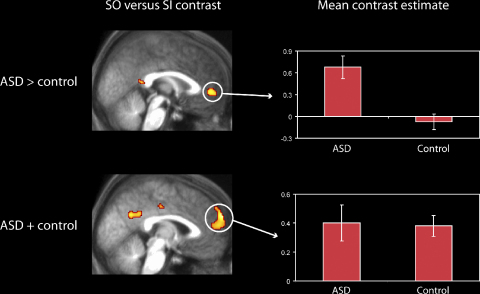
(Top) Regions showing significantly greater activation related to the contrast of SO > SI in the ASD than the control group. (Bottom) Regions showing activation in the contrast of SO > SI when the two groups are pooled. Results are plotted on a sagittal slice of the participants’ mean normalized structural scan (top: *x* = 0, bottom: *x* = 2, so that each slice displays the relevant peak voxel). Mean contrast estimates are plotted at the peak BA 10 voxel for each contrast (top: 0, 48, 2; bottom: 2, 64, 28) separately for the ASD and control groups. Error bars indicate standard errors.

Inspection of Tables 4 and 5 suggests differences between the ASD and control groups not only in the overall level of medial rostral PFC activation associated with the SO > SI contrast, but also in the location of activation peaks. Specifically, the peak medial rostral PFC activations appear to be relatively caudal in the ASD group, compared with the control group. In order to test for such differences, the peak medial rostral PFC co-ordinate for the SO > SI contrast was extracted individually for each participant (see Gilbert, Williamson, et al., 2007, for a similar approach). Medial rostral PFC was defined here as −8 ≤ *x* ≤ 8, *y* > 40, −12 ≤ *z* ≤ 30, as in our previous study (Gilbert, Williamson, et al., 2007). Analysis of these data confirmed that peak co-ordinates were indeed more caudal in the ASD group than the control group (ASD: mean *y* = 52.9; control: mean *y* = 58.4; *F*(1, 31) = 4.8, *p* < .05). However, a similar analysis of the medial rostral PFC peaks associated with the Baseline > Random contrast did not produce a significant group difference (ASD: mean *y* = 53.3; control: mean *y* = 55.1; *F* < 1). The results from the SO > SI contrast are illustrated in Fig. 6, which displays regions showing greater activity associated with the SO > SI contrast in the ASD than the control group, along with results from the SO > SI contrast in an analysis where the two groups were combined. It can be seen that the more caudal medial PFC region activated in the subtraction between the groups is activated in the ASD but not in the control group. By contrast, the more rostral medial PFC region activated when the groups were combined shows similar levels of activity in the two groups. It therefore seems likely that the ASD group showed more widespread activity related to the SO > SI contrast than the control group.

## 3 Discussion

In this study we administered two tests of executive function to a group of high-functioning participants with ASD and an age- and IQ-matched control group: a “classical” test of random keypress generation, and a new test involving selection between stimulus-oriented and stimulus-independent thought (“alphabet test”). Behavioural performance was similar in the two groups. In both tasks, the two groups activated partially overlapping brain regions, including lateral prefrontal and parietal cortex, which showed relatively high activity during the more demanding conditions (random sequence generation, and stimulus-independent cognition), and medial rostral prefrontal cortex, which showed relatively low activity during such conditions, compared with baseline and stimulus-oriented conditions. However, task-specific between-group differences were also observed in the neuroimaging data. In the random generation task, between-group differences were observed in the cerebellum and lateral temporal cortex. The alphabet test revealed more extensive between-group differences. In the contrast of stimulus-oriented versus stimulus-independent phases, the ASD group showed greater activity in medial prefrontal, temporal, parietal, and cerebellar regions, whereas the control group showed greater activity in distinct occipital and parietal regions. In addition, there was evidence that in the alphabet test the groups differed not only in the overall level of activity in medial rostral PFC, but also in the location of the activation peaks, with activation peaks in the ASD group being caudal to those in the control group.

These results suggest that even within the same participants, performance of different executive function tasks may be associated with functional abnormalities in different brain regions in high-functioning participants with ASD. Of course, it remains to be seen whether such results would generalise to less high-functioning participants. However, such results underline the importance of examining task-specific effects within the domain of executive function, rather than considering any single task as an exhaustive indicator of this domain. Furthermore, these results suggest that new tests of executive function, requiring selection between stimulus-oriented and stimulus-independent thought, may be particularly sensitive to atypical recruitment of rostral PFC, and functional organisation of this region, in participants with ASD. Below, we consider the results from the two tasks in greater depth. However, in order to interpret the present results, it is important first to discuss the issue of “activation” versus “deactivation” in functional neuroimaging studies.

### 3.1 “Activation” versus “deactivation” in functional neuroimaging

In the present study, each task consisted of a relatively demanding condition (random generation or stimulus-independent cognition), involving a relatively indirect link between stimuli and responses, and a less demanding condition (sequence generation or stimulus-oriented cognition) where responses were driven in a more direct manner by environmental events. In both tasks, and both groups, some brain regions showed greater activity in the more demanding conditions, whilst other brain regions – notably medial rostral PFC – showed greater activity in the less demanding conditions. The functional role of brain regions that show enhanced activity during low-demand conditions (i.e. that are “deactivated” by relatively demanding conditions) is presently a matter of considerable debate (e.g. Gilbert, Dumontheil, Simons, Frith, & Burgess, 2007; Morcom & Fletcher, 2007). According to some authors, such brain regions may support task-unrelated processes (e.g. “daydreaming”) that are suspended during more demanding conditions (e.g. McKiernan, Kaufman, Kucera-Thompson, & Binder, 2003). However, other authors have suggested that these brain regions play a functional role, for example by promoting attention towards the external environment in low-demand tasks (e.g. Gilbert, Simons, et al., 2006; Gilbert, Williamson, et al., 2007). This hypothesis is supported by neuroimaging investigations of the relationship between BOLD signal in such regions and behavioural performance (Gilbert, Simons, et al., 2006; Gilbert, Spengler, Simons, Frith, & Burgess, 2006), along with neuropsychological studies showing impaired performance on low-demand tasks (e.g. simple RT) in patients with damage to these regions (Stuss et al., 2005). We therefore consider all significant BOLD signal changes between conditions to be potentially noteworthy, rather than restricting analysis to just those regions showing greater activity in more demanding conditions.

A recent study by Kennedy, Redcay, and Courchesne (2006) suggested that brain regions exhibiting greater signal during relatively low-demand conditions, including medial rostral PFC, show attenuated activity in such conditions (or a ‘failure to deactivate’) in participants with autism spectrum disorders. In this study, participants either performed a demanding Stroop-like task (Bush et al., 1998) or were instructed to passively view a fixation cross. Whereas the control group exhibited greater signal in medial rostral PFC during fixation than the Stroop condition, no such effect was observed in the ASD group. The authors interpreted these findings in terms of task-unrelated cognitive processes (e.g. self-referential thought) during low-demand conditions in the control but not the ASD group. The present results corroborate the findings of Kennedy et al. (2006) in suggesting functional abnormalities of medial rostral PFC in ASD. However, our results do not fully support Kennedy et al.'s (2006) interpretation of their findings. In the present study, the ASD group showed increased medial rostral PFC activity in the low-demand versus high-demand conditions, so there was no evidence for a failure to deactivate in either task. Moreover, the difference in medial rostral PFC activity between stimulus-oriented and stimulus-independent conditions of the alphabet task was *greater* than the corresponding difference in the control group (i.e. an effect in the opposite direction to that observed by Kennedy et al., 2006).

In the terminology of Kennedy et al. (2006) this could be described as an *enhanced* deactivation in the SI relative to the SO condition, rather than a failure to deactivate. These results suggest that medial rostral PFC activity differs between participants with ASD and control participants in a task-dependent manner, rather than ASD participants always showing reduced deactivation of medial rostral PFC in high-demand conditions. However, it is not possible to compare the present results directly with those of Kennedy et al. (2006). The present study did not include a condition such as ‘rest’ or ‘fixation’, because activity observed in such conditions may be difficult to relate to underlying cognitive processes (Gilbert, Dumontheil, et al., 2007). It is therefore not possible to investigate whether the ASD group would have shown deactivation relative to such conditions (or not, as reported by Kennedy et al., 2006; see Morcom & Fletcher, 2007, for discussion of the merits and shortcomings of such low-level baseline tasks in functional neuroimaging studies).

### 3.2 Task-specific abnormalities in executive function

The present results are consistent with recent evidence suggesting an uneven profile of executive function abnormalities in autism spectrum disorders (Hill & Bird, 2006; Minshew et al., 1997). Rather than the two executive function tasks revealing consistent between-group differences, differences between the two groups were observed in different regions, depending on the task. Notably, only the new test of executive function (alphabet task) revealed frontal-lobe differences between the groups. Other functional imaging studies investigating participants with ASD have produced inconsistent results, with some studies indicating task-related increases in activity in participants with ASD (e.g. Schmitz et al., 2006), some indicating task-related decreases (e.g. Castelli et al., 2002; Luna et al., 2002), and others indicating a combination of task-related increases and decreases in different brain regions (e.g. Müller, Cauich, Rubio, Mizuno, & Courchesne, 2004). These studies have also reported between-group differences in a variety of brain regions. The present results suggest that this variability can be attributed not only to methodological differences between studies such as the use of different participant groups, but also to task-specific differences. Even within two tasks sensitive to executive function, between-task differences were observed in the present study, within the same group of participants (see also Schmitz et al., 2006, for a similar result within this domain).

In the random generation task, between-group differences were observed in the cerebellum. Along with structural change (Courchesne, 1997), this area has been shown to exhibit functional abnormalities in ASD in several previous studies, particularly those involving motor sequencing tasks (e.g. Allen, Müller, & Courchesne, 2004). Thus, the functional difference observed in the random generation task may have reflected the demands of this task to co-ordinate a sequence of random responses with the visual timing signal, rather than other aspects of the task (e.g. inhibition of prepotent response sequences, or switching between different random generation strategies).

In the alphabet task, the main region showing between-group differences was medial rostral PFC. Previous studies have indicated that this region plays a role in attentional selection between stimulus-oriented and stimulus-independent thought, in tasks such as the alphabet test which may be particularly sensitive to this form of attentional selection (e.g. Gilbert et al., 2005; Gilbert, Simons, et al., 2006; Gilbert, Williamson, et al., 2007). This region has also been implicated in multitasking and prospective memory (i.e. organising one's behaviour according to previously formed, internally represented intentions; e.g. Burgess, 2000; Burgess et al., 2000; Burgess, Quayle, & Frith, 2001; Burgess, Scott, & Frith, 2003; Burgess, Dumontheil, Gilbert, Okuda, et al., 2007; Simons, Schölvinck, Gilbert, Frith, & Burgess, 2006). This anatomical link, along with the present evidence for functional abnormalities in this region in ASD, suggests that dysfunction in rostral PFC may, at least in part, underlie deficits seen in ASD in recent tests of executive function that involve multitasking and prospective memory, such as the Six Element Test (Hill & Bird, 2006; Shallice & Burgess, 1991). In addition, these results are consistent with previous suggestions that multitasking and prospective memory are reliant on attentional selection between stimulus-oriented and stimulus-independent information (Burgess et al., 2003; Burgess, Dumontheil, Gilbert, Okuda, et al., 2007).

Whereas medial rostral PFC showed greater activity related to the stimulus-oriented versus stimulus-independent contrast in the ASD group, the control group showed greater activity primarily in bilateral occipital cortex. This suggests that the control group were able to modulate activity in early visual cortex according to the attentional demands of the task to a greater degree than the ASD group. The stimuli were matched between the two conditions, suggesting attentional modulation rather than an effect of stimulus-category. This finding is consistent with the suggestion of functional underconnectivity in ASD (e.g. Bird, Catmur, Silani, Frith, & Frith, 2006; Castelli et al., 2002; Frith, 2003; Just et al., 2007), leading to a decrease in top-down modulation of sensory areas according to attentional demands. Further evidence consistent with such an account comes from the finding of a group difference in lateral temporal cortex in the random generation task. Jahanshahi et al. (2000) argue that activity in lateral temporal regions is suppressed by lateral prefrontal cortex in order to prevent stereotyped behaviour in random generation tasks. The ASD group's reduced difference between baseline and random-generation conditions in lateral temporal cortex may reflect a reduction in such top-down modulation.

### 3.3 Functional organisation of medial prefrontal cortex

Recent studies have indicated considerable functional specialisation within rostral PFC (Gilbert, Spengler, Simons, Steele, et al., 2006; Gilbert, Williamson, et al., 2007). Two functions that have been associated with medial rostral PFC are stimulus-oriented attention (e.g. Gilbert et al., 2005) and mentalizing (Frith & Frith, 2003). However, a recent meta-analysis (Gilbert, Spengler, Simons, Steele, et al., 2006) suggested that rostral PFC activation peaks associated with mentalizing were significantly posterior (mean *y* = 53) to those associated with other cognitive domains, such as multiple-task co-ordination (including prospective memory; mean *y* = 61), even though the mean co-ordinates of activation peaks were just a few millimetres apart. This suggestion of functional specialisation was confirmed in a study that crossed the factors of attention (stimulus-oriented versus stimulus-independent) and mentalizing (mentalizing versus non-mentalizing) in a factorial design (Gilbert, Williamson, et al., 2007). Activation peaks associated with the mentalizing manipulation were found to be significantly posterior to those from the attention manipulation, within the same participants. Thus, even though medial rostral PFC has been implicated in both mentalizing and multitasking, it seems that these two functions may depend on separable subregions. In this light, it is interesting to note that the present study indicated differences between the ASD and control groups not only in the overall level of medial rostral PFC activity associated with the stimulus-oriented versus stimulus-independent comparison, but also in the location of activation peaks. Activation peaks from the ASD group were significantly posterior to those from the control group, suggesting functional re-organisation of medial rostral PFC in ASD. Moreover, the region of medial rostral PFC activated in the ASD group was more akin to the region previously implicated in mentalizing (Gilbert, Spengler, Simons, Steele, et al., 2006; Gilbert, Williamson, et al., 2007; Simons, Henson, Gilbert, & Fletcher, 2008) than the region activated in the control group. This raises the possibility that participants with ASD may recruit brain regions typically associated with mentalizing for the performance of other tasks (for further evidence of functional brain reorganisation in ASD, see Pierce, Müller, Ambrose, Allen, & Courchesne, 2001, who suggest that participants with ASD use atypical brain regions for face perception). Consistent with this hypothesis, other regions showing enhanced activity in the ASD group associated with the stimulus-oriented versus stimulus-independent contrast included the temporal pole and amygdala, both of which have been previously implicated in social cognition (Adolphs, 2006; Frith & Frith, 2003).

## Figures and Tables

**Table 1:** Brain regions showing significantly greater activity in the random generation than baseline condition

Region	BA	Hemisphere	*x*	*y*	*z*	*Z*_max_	*N* voxels
ASD							
Lateral PFC	10	L	−38	54	16	4.01	165
	10	R	48	48	16	3.86	183
	9/46	L	−44	42	28	3.35	63
	47	R	36	26	0	4.23	295
	47	L	−38	26	2	3.38	59
Anterior cingulate	32	L	−6	20	40	4.81	1835
Lateral PFC	47	L	−48	16	−6	3.94	143
	44	R	50	14	10	3.29	24
	44	L	−52	12	18	4.04	196
	9	R	56	12	44	3.70	123
Lateral premotor cortex	6	R	30	0	58	4.95	1610
Lateral parietal cortex	40	R	60	−24	46	4.01	171
	40	R	44	−40	60	3.19	18
	40	L	−46	−42	44	4.29	322
Cerebellum	–	R	38	−46	−38	4.39	377
	–	L	−34	−56	−32	4.55	698
Medial parietal cortex	7	R	16	−58	70	4.09	221
	7	L	−14	−58	64	3.59	60
	7	R	20	−66	42	3.38	10
	7	L	−8	−74	44	3.30	25

Control							
Lateral PFC	10	R	46	54	−4	3.27	15
	46	R	36	32	24	3.79	503
	47	R	32	30	−2	3.54	186
	47	L	−22	28	−6	3.72	10
Insula	13	L	−32	18	10	3.41	119
Lateral PFC	44	L	−46	6	28	3.12	10
Premotor cortex	6	R	24	−4	42	4.73	2360
	6	L	−24	−8	42	4.20	550
Thalamus	–	R	14	−8	−2	3.21	7
Lateral parietal cortex	7	R	24	−38	66	3.54	9
	40	L	−46	−38	50	3.42	156
	7	R	16	−44	72	3.29	14
	40	R	46	−46	40	4.10	745
Cerebellum	–	L	−40	−48	−46	3.88	94
Medial parietal cortex	7	L	−26	−48	40	3.24	19
	7	L	−10	−66	60	3.49	73
	7	R	14	−72	56	4.02	298

*Note*. Co-ordinates refer to the Montreal Neurological Institute reference brain. Brodmann areas (BA) are approximate. L = left, R = right. PFC = prefrontal cortex.

**Table 2:** Brain regions showing significantly greater activity in the baseline than random generation condition

	BA	Hemisphere	*x*	*y*	*z*	*Z*_max_	*N* voxels
ASD							
Medial PFC	10	R	6	50	−8	3.40	82
	24	L	−14	32	6	3.32	8
Medial temporal lobe	36	R	26	−32	−20	3.32	18
Posterior cingulate	31	L	−18	−40	28	3.55	10
Lateral parietal cortex	39	L	−38	−52	26	3.28	8
Posterior cingulate/precuneus	23	B	0	−52	24	3.23	32
	31	L	−12	−62	30	3.46	58
Lateral occipital cortex	19	R	36	−92	2	3.44	8

Control							
Medial PFC	8	B	0	50	50	3.55	95
	8	R	18	48	46	3.43	23
	10/11	B	4	46	−10	3.79	216
Lateral PFC	8	L	−32	30	52	3.47	14
Medial PFC	24	B	2	24	−2	3.40	20
Lateral temporal cortex	38	L	−36	22	−32	3.47	30
	20	R	38	−10	−36	4.27	248
Medial temporal lobe	35	R	20	−16	−34	3.64	21
Lateral temporal cortex	20	R	48	−20	−18	3.50	38
	21	L	−52	−24	−12	3.91	126
	36	R	40	−32	−26	4.58	388
	36	L	−32	−32	−26	3.89	322
Cerebellum	–	L	−12	−40	−26	3.18	26
Posterior cingulate	30	L	−6	−54	6	3.16	7
	23	R	10	−60	14	3.36	42
Lateral occipital cortex	37	L	−42	−62	−16	3.43	27
Lateral parietal cortex	39	R	60	−62	26	3.30	10
Posterior cingulate/precuneus	31	L	−10	−68	18	3.61	323
Lateral parietal cortex	39	L	−52	−70	34	3.97	316
Lateral occipital cortex	19	R	50	−78	20	3.61	11

*Note*. Co-ordinates refer to the Montreal Neurological Institute reference brain. Brodmann areas (BA) are approximate. L = left, R = right, B = bilateral. PFC = prefrontal cortex.

**Table 3:** Brain regions showing significantly greater activity in the stimulus-independent than stimulus-oriented condition of the alphabet task

Region	BA	Hemisphere	*x*	*y*	*z*	*Z*_max_	*N* voxels
ASD							
Lateral PFC	46	L	−50	44	6	3.63	21
	10	R	38	42	26	3.79	34
Medial PFC	8	L	−2	24	50	3.88	140
Lateral PFC	47	L	−36	22	0	3.61	102
	47	R	30	20	−8	3.27	7
	44	L	−44	12	26	4.71	443
Premotor cortex	6	L	−36	8	54	3.55	14
Lateral PFC	9/44	L	−34	6	34	4.47	161
Superior parietal cortex	7	L	−26	−50	34	3.86	22
	7	L	−36	−60	54	4.20	216
	7	R	10	−64	52	3.86	79

Control							
Lateral PFC	10	R	24	56	4	3.77	95
	10/46	R	34	48	22	3.32	8
	10	L	−36	44	0	3.93	101
	9	L	−26	38	40	3.98	12
	10/46	R	44	38	26	3.54	66
Medial PFC	9/32	L	−4	38	38	3.33	13
Lateral PFC	47	R	34	24	−4	3.67	157
Medial PFC	32	B	2	22	36	4.39	621
Lateral PFC	8	R	28	20	50	3.73	111
	44/45	L	−52	18	16	3.17	6
Premotor cortex	6	R	40	6	46	3.65	55
	6	L	−40	4	42	4.57	908
Thalamus	–	B	0	−8	4	3.52	12
Lateral parietal cortex	40	L	−50	−36	50	3.83	38
	40	R	52	−36	46	3.77	39
	40	R	38	−56	46	3.48	54
Cerebellum	–	R	10	−56	−2	3.40	17
Superior parietal cortex	7	L	−10	−68	54	3.39	9
	7	L	−28	−70	54	3.63	133
Medial occipital cortex	18	R	8	−84	−4	3.60	27

*Note*. Co-ordinates refer to the Montreal Neurological Institute reference brain. Brodmann areas (BA) are approximate. L = left, R = right, B = bilateral. PFC = prefrontal cortex.

**Table 4:** Brain regions showing significantly greater activity in the stimulus-oriented than stimulus-independent condition of the alphabet task

Region	BA	Hemisphere	*x*	*y*	*z*	*Z*_max_	*N* voxels
ASD							
Medial PFC	9	R	10	56	36	3.98	129
	10	L	−6	56	18	3.42	62
	10	L	−12	50	0	4.21	221
	9	L	−14	38	36	3.76	70
Lateral temporal cortex	38	R	52	12	−26	3.26	9
	21	L	−50	2	−30	3.83	96
Cingulate gyrus	24	R	2	−2	36	3.41	7
Medial temporal lobe	–	R	32	−12	−18	3.77	71
Lateral temporal cortex	20	L	−64	−14	−24	4.03	24
Cingulate grus	24/31	L	−6	−18	44	3.73	149
Lateral parietal cortex	40	R	66	−26	24	3.44	8
	40	L	−38	−30	62	3.41	7
	40	R	32	−44	60	3.32	14
Cerebellum	–	R	20	−44	−20	3.28	11
	–	R	22	−52	−22	3.26	8
	–	L	−28	−62	−50	3.60	41
	–	R	48	−64	−38	3.60	9
	–	R	34	−72	−40	3.53	16

Control							
Medial PFC	10/9	B	4	64	30	4.19	152
Lateral parietal cortex	40	L	−64	−8	26	3.25	7
	40	R	66	−16	28	3.52	22
Lateral occipital cortex	18/19	R	26	−92	6	3.89	403
	18/19	L	−22	−100	14	3.87	670

*Note*. Co-ordinates refer to the Montreal Neurological Institute reference brain. Brodmann areas (BA) are approximate. L = left, R = right, B = bilateral. PFC = prefrontal cortex.

**Table 5:** Brain regions showing significant differences in activity related to the SO > SI contrast between the ASD and control groups

Region	BA	Hemisphere	*x*	*y*	*z*	*Z*_max_	*N* voxels
ASD > Control							
Medial PFC	9	R	10	54	36	3.94	159
	10	B	0	48	2	3.62	130
	10	L	−16	44	24	3.33	8
	9	L	−12	36	38	4.44	144
	32	R	10	30	26	3.85	27
Temporal pole	38	R	36	22	−32	3.18	8
Medial temporal cortex	28	R	26	8	−22	3.80	79
Temporal pole	21/38	L	−58	4	−24	3.64	49
Amygdala	–	R	26	−6	−16	3.34	25
Premotor cortex	6	L	−22	−8	48	3.61	14
Posterior cingulate	31	R	8	−28	46	3.29	7
Precentral gyrus	4	R	14	−34	66	3.22	7
Lateral temporal cortex	22	R	40	−40	6	3.29	8
Lateral parietal cortex	40	R	28	−42	60	3.33	17
Posterior cingulate	30	R	2	−46	16	3.37	19
Cerebellum	–	R	18	−48	−20	3.45	31
Occipito-temporal cortex	37	L	−38	−56	4	3.36	11

Control > ASD							
Medial parietal cortex	7	R	12	−62	46	3.36	13
Medial occipital cortex	18	R	16	−96	16	3.58	22

*Note*. Co-ordinates refer to the Montreal Neurological Institute reference brain. Brodmann areas (BA) are approximate. L = left, R = right, B = bilateral. PFC = prefrontal cortex.
